# Feasibility of a Home-Based Cognitive-Physical Exercise Program in Patients With Chronic Obstructive Pulmonary Disease: Protocol for a Feasibility and Pilot Randomized Controlled Trial

**DOI:** 10.2196/48666

**Published:** 2023-07-12

**Authors:** Dmitry Rozenberg, Josh Shore, Encarna Camacho Perez, Sahar Nourouzpour, Megha Ibrahim Masthan, Daniel Santa Mina, Jennifer L Campos, Ella Huszti, Robin Green, Mohammad Hashim Khan, Ambrose Lau, David Gold, Matthew B Stanbrook, W Darlene Reid

**Affiliations:** 1 Respirology and Lung Transplantation Toronto General Hospital Research Institute University Health Network Toronto, ON Canada; 2 Temerty Faculty of Medicine University of Toronto Toronto, ON Canada; 3 Division of Respirology University Health Network Toronto, ON Canada; 4 Faculty of Kinesiology and Physical Education University of Toronto Toronto, ON Canada; 5 Department of Anesthesia and Pain Management University Health Network Toronto, ON Canada; 6 Toronto Rehabilitation Institute (KITE) University Health Network Toronto, ON Canada; 7 Department of Psychology University of Toronto Toronto, ON Canada; 8 Biostatistics Research Unit University Health Network Toronto, ON Canada; 9 Krembil Neuroscience Centre, Toronto Western Hospital University Health Network Toronto, ON Canada; 10 Department of Physical Therapy University of Toronto Toronto, ON Canada; 11 Interdivisional Department of Critical Care Medicine University of Toronto Toronto, ON Canada

**Keywords:** pulmonary disease, chronic obstructive, telerehabilitation, physical exercise, cognitive training, randomized controlled trial, pilot study

## Abstract

**Background:**

Chronic obstructive pulmonary disease (COPD) is a progressive condition associated with physical and cognitive impairments contributing to difficulty in performing activities of daily living (ADLs) that require dual tasking (eg, walking and talking). Despite evidence showing that cognitive decline occurs among patients with COPD and may contribute to functional limitations and decreased health-related quality of life (HRQL), pulmonary rehabilitation continues to focus mainly on physical training (ie, aerobic and strength exercises). An integrated cognitive and physical training program compared to physical training alone may be more effective in increasing dual-tasking ability among people living with COPD, leading to greater improvements in performance of ADLs and HRQL.

**Objective:**

The aims of this study are to evaluate the feasibility of an 8-week randomized controlled trial of home-based, cognitive-physical training versus physical training for patients with moderate to severe COPD and derive preliminary estimates of cognitive-physical training intervention efficacy on measures of physical and cognitive function, dual task performance, ADLs, and HRQL.

**Methods:**

A total of 24 participants with moderate to severe COPD will be recruited and randomized into cognitive-physical training or physical training. All participants will be prescribed an individualized home physical exercise program comprising 5 days of moderate-intensity aerobic exercise (30-50 minutes/session) and 2 days of whole-body strength training per week. The cognitive-physical training group will also perform cognitive training for approximately 60 minutes, 5 days per week via the BrainHQ platform (Posit Science Corporation). Participants will meet once weekly with an exercise professional (via videoconference) who will provide support by reviewing the progression of their training and addressing any queries. Feasibility will be assessed through the recruitment rate, program adherence, satisfaction, attrition, and safety. The intervention efficacy regarding dual task performance, physical function, ADLs, and HRQL will be evaluated at baseline and at 4 and 8 weeks. Descriptive statistics will be used to summarize intervention feasibility. Paired 2-tailed *t* tests and 2-tailed *t* tests will be used to compare the changes in the outcome measures over the 8-week study period within and between the 2 randomized groups, respectively.

**Results:**

Enrollment started in January 2022. It is estimated that the enrollment period will be 24 months long, with data collection to be completed by December 2023.

**Conclusions:**

A supervised home-based cognitive-physical training program may be an accessible intervention to improve dual-tasking ability in people living with COPD. Evaluating the feasibility and effect estimates is a critical first step to inform future clinical trials evaluating this approach and its effects on physical and cognitive function, ADL performance, and HRQL.

**Trial Registration:**

ClinicalTrials.gov NCT05140226; https://clinicaltrials.gov/ct2/show/NCT05140226

**International Registered Report Identifier (IRRID):**

DERR1-10.2196/48666

## Introduction

### Background

Chronic obstructive pulmonary disease (COPD) is a progressive respiratory condition characterized by chronic bronchitis and emphysema [1]. It is the third-leading cause of death worldwide [2] and is responsible for high levels of health care use because of frequent respiratory exacerbations [3]. Individuals with COPD experience significant airflow obstruction and dynamic hyperinflation of the lungs, leading to symptoms of dyspnea, sputum production, chronic cough, and fatigue [1]. Skeletal muscle dysfunction, comprising decreased muscular strength and endurance, is also a common consequence of COPD and is driven by a combination of several mechanisms, including muscle atrophy, altered fiber type, and impaired metabolism [4]. Collectively, these changes contribute to diminished exercise capacity, limitations in activities of daily living (ADLs), and reduced health-related quality of life (HRQL) among individuals with COPD [5].

### Cognitive Impairment in COPD

A growing body of research has demonstrated the presence of limitations across several domains of cognition among individuals with COPD [6]. Decreases in attention, memory, and visual-motor processing have been commonly reported [6-8]. The underlying mechanisms of cognitive impairment among individuals with COPD are complex and have been attributed in part to neural tissue hypoxemia and systemic inflammation [9,10].

Recent studies have indicated that COPD is associated with impaired dual-tasking performance [11,12]. Dual tasking, the performance of 2 tasks simultaneously, is an important aspect of executive function and is often required for the performance of routine daily activities, such as walking and talking. The performance of a cognitive task (eg, spelling words backward) while carrying out a physical task (ie, usual-paced walking) can result in decrements in cognitive or physical performance (eg, walking speed or distance) owing to competing attentional demands. Dual task decrements (difference between dual and single task performance) are often more pronounced in those with existing cognitive impairments or neurological conditions compared with those without these disorders [13,14]. Indeed, increased dual task decrements for both motor [12,15] and cognitive [16] performance have been found among people with COPD compared with healthy individuals [17]. There is emerging evidence indicating that cognitive-motor dual task performance impairment among patients with COPD is related to alterations in markers of prefrontal cortex neural activity and inefficiency, which may impede the executive function needed to *plan one’s day* [11,12,17]. Thus, combined cognitive-physical training may be a good strategy to improve neural efficiency in patients with COPD who may have a range of executive functional capacities. Cognitive training targeting attention, memory, executive functioning, and speed of processing when combined with physical exercise training may provide the neuroplasticity to derive synergistic benefits on the performance of ADLs and physical function [18-20].

### Traditional Pulmonary Rehabilitation

Pulmonary rehabilitation is considered a primary management approach for moderate to severe COPD and has been shown to reduce dyspnea and increase exercise capacity, leading to improvements in daily function and HRQL among individuals with COPD [21]. To date, pulmonary rehabilitation strategies have typically focused on education (eg, medication management, breathing techniques, and stress reduction) and physical training (ie, aerobic and strength exercises) [21,22]. Currently, cognitive training is not included in most pulmonary rehabilitation programs despite evidence that cognitive impairments are common among individuals with COPD and are associated with greater functional limitations, decreased HRQL, and increased health care use in this population [8,23].

### Home-Based Pulmonary Rehabilitation

Several barriers limit engagement in pulmonary rehabilitation for people with COPD, including factors related to the environment (eg, travel and wait times), knowledge (eg, referral processes), and beliefs (eg, perceived benefits) [24]. Recently, accessibility has been further complicated by the COVID-19 pandemic. For these reasons, there is increasing interest in home-based pulmonary rehabilitation programs as an alternative strategy for COPD management compared with center-based pulmonary rehabilitation programs [25-27]. Several studies have demonstrated the safety and feasibility of remote telerehabilitation interventions for patients with COPD, with high levels of compliance with home training and no adverse events [28-30]. There is meta-analytic evidence from systematic reviews also indicating that the effects of home-based pulmonary rehabilitation interventions are similar to those of traditional outpatient programs for outcomes including dyspnea [26,27], exercise capacity [26,31], and HRQL [26,31], potentially offering increased accessibility to those with travel restrictions [32]. However, most studies on telerehabilitation for COPD have required study assessments to be conducted in person, and none have included remote assessment of functional testing, cognitive assessments, or dual task performance, a knowledge gap that this study hopes to address [27].

### Study Purpose

The specific aims of this study are to evaluate the feasibility (ie, recruitment rate, program adherence, attrition, safety, and participant satisfaction) of an 8-week individualized, home-based cognitive-physical training program for patients with moderate to severe COPD and derive preliminary estimates of cognitive-physical training intervention efficacy on measures of cognitive and physical function, dual task performance, ADL, and HRQL. We hypothesize that it will be feasible to safely recruit patients with COPD into a home-based cognitive-physical training program with 75% adherence and high satisfaction ratings with the prescribed training. We also hypothesize that the cognitive-physical training group will demonstrate greater beneficial effects on cognitive function, dual task performance, ADL, and HRQL compared with the physical training group.

## Methods

### Design

This is a pilot randomized controlled trial (RCT) to assess the feasibility of a full-scale, adequately powered RCT evaluating a home-based combined cognitive-physical training program for patients with moderate to severe COPD. The study takes place at the University Health Network (UHN) in Toronto, Ontario, Canada, but has been expanded to include recruitment from community respirology clinics not affiliated with the UHN.

### Ethics Approval and Informed Consent

Research ethics approval was obtained through the research ethics board of the UHN (study ID: 21-5336), and the study has been registered at ClinicalTrials.gov (NCT05140226). Informed consent will be obtained from participants before study enrollment.

### Participants

The study sample will consist of older adults (aged ≥50 years) with a clinical and spirometric diagnosis of moderate to severe COPD (postbronchodilator forced expiratory volume in the first second [FEV_1_]/forced vital capacity of <0.70 [or lower limit of normal of FEV1/forced vital capacity] and FEV_1_=30%-79%). Participants will be required to have access to a reliable internet connection and be able to mobilize independently and safely with or without a walking aid. Exclusion criteria include the following: (1) current participation in supervised in-person or an internet-based pulmonary rehabilitation program; (2) COPD exacerbation in the previous 3 months to ensure clinical stability; (3) presence of cardiovascular, neurological, or musculoskeletal conditions that could interfere with the safe performance of study procedures (eg, unstable coronary artery disease, heart failure, stroke, myopathy, or moderate to severe arthritis); (4) severe cognitive impairment ascertained through chart review or self-report (eg, severe dementia or inability to perform ADLs independently); (5) medical instability (eg, hepatic dysfunction, metabolic abnormality, or active infection); (6) overt psychiatric disorder or substance abuse; (7) current use of home oxygen therapy; (8) insufficient English fluency to carry out testing and training; and (9) being listed for lung transplantation.

### Recruitment and Screening

Participants will be recruited during routine visits to outpatient respirology clinics at UHN in Toronto, Ontario, Canada, and local respirology clinics. Clinic personnel within the circle of care will introduce the study to patients with COPD and ask if they are interested in participating. If interested, a member of the research team will then contact the potential participant to screen for eligibility criteria, review study procedures, and obtain informed consent. The study recruitment poster will also be provided to medical personnel at local respirology clinics who will introduce the study to patients with COPD and instruct them to contact the research team if they are interested in pursuing study participation.

### Study Timeline

**Figure 1:**
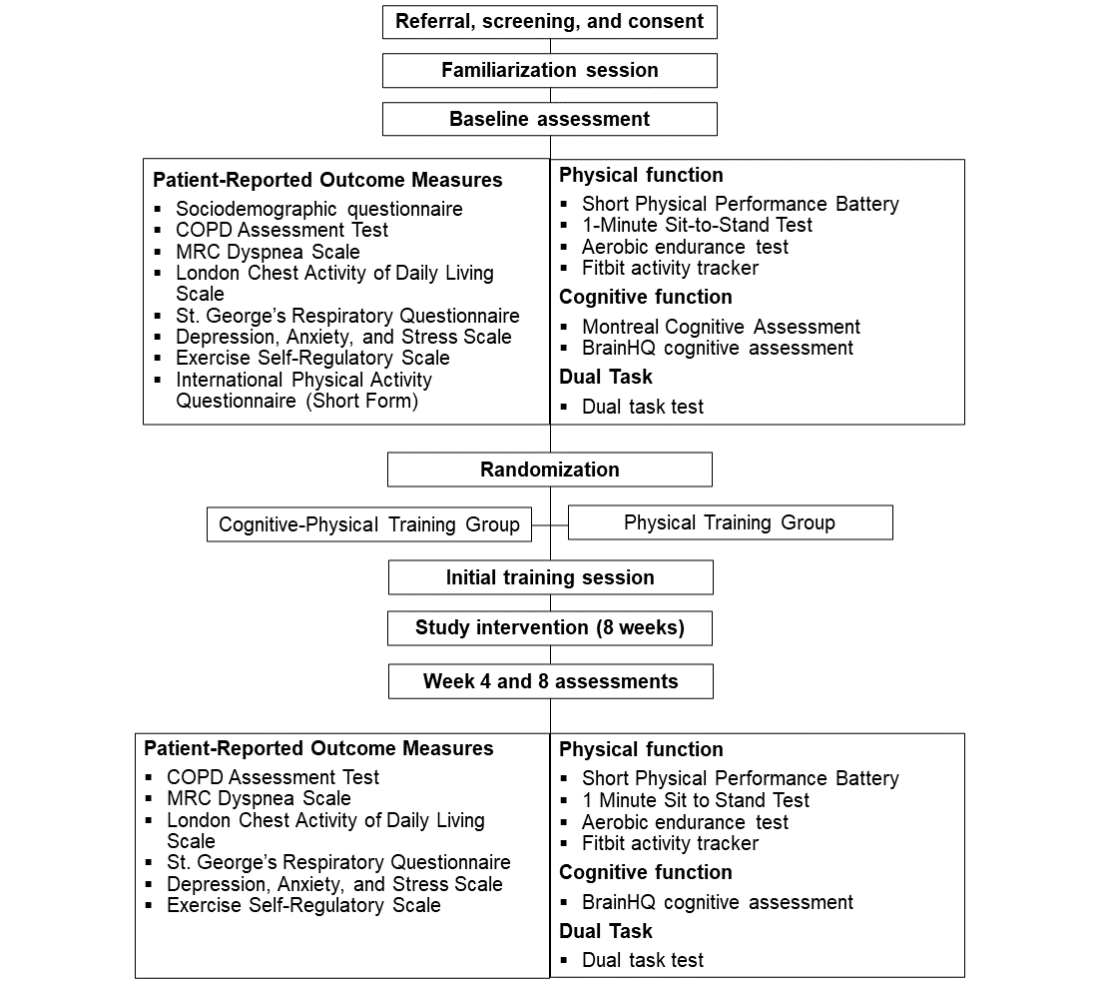
Study timeline. COPD: chronic obstructive pulmonary disease; MRC: Medical Research Council.

The study timeline is shown in Figure 1. After completing consent procedures, participants will receive a package by mail containing the following study equipment: Fitbit Charge 4 device (Fitbit Inc), foot peddler (VANGONA D2 pedal exerciser), pulse oximeter, resistance bands (Thera-Band), measuring tape, and exercise manual. They will also receive an email with instructions on how to access the internet-based appointment platform (Microsoft Teams; Microsoft Corp) on their devices. Following receipt of study materials, participants in both groups will be booked for a 45-minute videoconferencing familiarization session. During the familiarization session, participants will be introduced to the study equipment and software, their safety in performing remote physical testing will be assessed, and study assessment procedures will be explained. Furthermore, appropriate equipment setup for remote testing will be determined, and participants will have the opportunity to practice the physical assessment tests. After the familiarization session, participants will be booked for the *baseline assessment* session (1 hour) to complete the assessment measures described in the following sections.

### Randomization

After the baseline assessment, participants will be randomized to either the cognitive-physical training (intervention) group or the physical training (control) group using a 1:1 ratio. Randomization will be performed using shuffled opaque envelopes containing the group assignment. Given the pilot stage of this research and the nature of exercise interventions, group allocation will be known to the research team, exercise professionals, and study participants.

### Intervention Groups

#### Summary

After randomization, participants in both groups will complete an initial training session (1 hour) to introduce the intervention. The initial training session will consist of a health and exercise history assessment, tailored exercise prescription, goal setting, and software account setup, as described in the following sections. Both groups will also receive weekly videoconferencing calls (30-60 minutes) from the study team via Microsoft Teams. Weekly sessions will involve a review of training and any adverse events, progression or adaptation of the program as needed, and troubleshooting of any difficulties encountered by the participant. Participants will also have the option of scheduling additional sessions with the study team during the week if further support is needed.

#### Physical Training Group

##### Overview

Participants in the physical training group will perform 8 weeks of home-based physical exercise training as per the current standard of care for pulmonary rehabilitation [22,33]. The physical training program was developed according to established guidelines for pulmonary rehabilitation [22,33] and includes both aerobic and strength training exercises that will be individually tailored to the specific needs and physical condition of each participant. Table 1 summarizes the physical exercise training program parameters.

**Table 1:** Physical exercise training parameters.

Modality and frequency	Intensity	Time	Type	Progression
Aerobic: 5 days/week	Moderate to vigorous (target Borg RPE^a^ score of 4-6/10) 60%-85% of symptom-limited maximum heart rate	30-50 minutes Either continuous or interval training	Foot peddler	Gradual progression of training duration Increase pedaling speed (revolutions/minute) or resistance Increase target heart rate or RPE score
Strength: 2 days/week	Target Borg RPE score of 4-6/10	6 exercises 1-3 sets 10-12 repetitions	Resistance band exercises: band pull-aparts, seated bicep curls, seated rowing, and seated tricep kicks Body weight exercises: chair sit-to-stand exercises and calf raises	Resistance intensity will be increased when prescribed exercises can be completed without difficulty to maintain moderate exertion Increase in resistance band tension Increase in number of sets or repetitions

^a^RPE: Rating of Perceived Exertion.

Individualized progressive exercise prescriptions will be developed by an exercise professional and supported using a web application and mobile app (Physiotec) that allows for customizable exercise prescriptions with written instructions, video tutorials, and self-reported tracking of exercise completion. The exercise program will be reviewed, revised, and progressed as appropriate based on participants’ needs during the weekly check-ins with the exercise professional. This will involve reviewing available data from Fitbit and self-report records from the participant, including pulse oximeter recordings.

Participants will be encouraged to include a warm-up and a cooldown period as part of their exercise training sessions. The warm-up will be a 5-minute routine consisting of mobility and breathing exercises to be implemented before starting physical activity. The cooldown at the end of each training session will comprise neck, shoulder, and leg stretches. See Table 2 for examples of warm-up and cooldown exercises.

**Table 2:** Summary of warm-up and cooldown exercises.

Exercise		Intensity	Time	Type
**Warm-up (5 minutes)**				
	Breathing	As tolerated Gentle breathing exercises	Approximately 3 minutes	Pursed-lip breathing
	Mobility	Gentle mobility exercises as tolerated by the participants’ range of motion while combining with the rhythm of breathing	5 exercises 1 set of 10 repetitions	Neck mobility (chin tuck and head to the sides) Shoulder circles Arms reach up and reach down (touch ankles) Knee extension and flexion (performed while sitting in a chair)
**Cool down (5 minutes)**				
	Stretching	Gentle stretching exercises as tolerated by the participants’ range of motion	3 exercises 1 set of 2 repetitions for each exercise Hold each exercise for 10 seconds	Chest stretching in chair Back stretching in chair Leg stretching in chair

##### Aerobic Training

Aerobic training will consist of using a foot peddler (VANGONA D2 pedal exerciser) provided by the research team (Table 1). Participants will be asked to perform aerobic training 5 times per week for a minimum of 30 minutes (up to 50 minutes) at a moderate to vigorous intensity (level 4-6 out of 10 on the Borg Rating of Perceived Exertion [RPE] Scale). Aerobic exercise may be performed in intervals (eg, 5 minutes of pedaling and 2 minutes of rest) or completed in shorter bouts throughout the day if participants cannot tolerate 30 minutes continuously. Exercise duration and intensity will be adapted to the participants’ level of dyspnea and fatigue. Aerobic training will progress under the guidance of the exercise professional by increasing duration, cycling speed, and resistance.

##### Strength Training

Strength training will be performed 2 times per week using resistance bands (provided by the research team), the participants’ own body weight, and household items (eg, chairs). Participants will be prescribed 1 to 3 sets of 10 to 12 repetitions for 6 strength exercises targeting large muscle groups such as the chest, back, arms, shoulders, and legs. Exercises may include band pull-aparts, seated rowing, bicep curls, triceps extensions, sit-to-stand exercises, and calf raises. Specific exercises and the number of sets and repetitions will be tailored to suit the individual needs of each participant, with a target intensity level of 4 to 6 on the Borg RPE Scale. Strength training will be performed under the guidance of the exercise professional by increasing the number of sets or repetitions or by increasing the tension in the resistance bands.

##### Safety Precautions for Remote Training

Participants will be given a personalized exercise program supervised by a qualified exercise professional. The exercise professional will review important considerations for home exercise, such as maintaining an open training environment free from tripping hazards (eg, rugs, clutter, and pets), the suitability of exercise equipment setup, and appropriate exercise attire (eg, footwear). Before beginning the remote exercise sessions, the exercise professional will perform a safety check to determine the appropriateness of proceeding with testing or training. The safety check will involve an objective assessment of oxygen saturation, heart rate, and blood pressure (if available) and a subjective assessment of self-reported symptoms (eg, breathlessness, dizziness, and chest pain). The research team will also collect participants’ home addresses, telephone numbers, and emergency contact information to be used in the event of a medical emergency during internet-based interactions.

Participants will be instructed to monitor their oxygen saturation (pulse oximeter), heart rate (pulse oximeter or Fitbit), and subjective level of exertion (Borg RPE Scale) and breathlessness (Borg Dyspnea Scale) while exercising using printed scales. They will be advised to lower their intensity level (ie, decrease cycling speed or resistance) if they reach a score of ≥7 on the Borg Dyspnea or RPE Scales, if their oxygen saturation drops to <85%, or if they experience any other symptoms (eg, light-headedness). They will be asked to track any adverse events using the training diary and encouraged to contact a member of the research team immediately upon noticing any adverse events. If participants experience chest pain or discomfort (uncomfortable feeling of pressure, pain, squeezing, or heaviness in the chest spreading to the shoulder, arms, neck, and back), they will be instructed to immediately stop exercising, rest, and seek emergency medical assistance if the symptoms persist beyond 5 minutes. The training program will be monitored by the exercise professional via weekly internet-based appointments involving a review of physiological exercise parameters (heart rate and oxygen saturation) from the Fitbit data and participant self-reported records. Participants will also be able to contact the exercise professional and study team at any time if questions or concerns arise.

If a participant communicates a medically concerning adverse event or contraindication to exercise (eg, cardiovascular event), the program will be suspended until medical clearance to resume the exercise program is obtained from the participant’s physician and approved by the principal investigator. Participants who experience a COPD exacerbation during the study will be instructed to temporarily discontinue their training program. They will be reassessed by the principal investigator and exercise professional within 2 weeks for their ability to resume the study.

#### Cognitive-Physical Training Group

##### Overview

Participants in the cognitive-physical training group will perform 8 weeks of home-based web-based cognitive training in addition to physical exercise training.

##### Physical Exercise Training

Physical training, including aerobic and strength exercises identical to those in the physical training group, will be performed a minimum of 5 days per week, as described previously.

##### Cognitive Training

Cognitive training will be performed using the web-based BrainHQ training platform (Posit Science Corporation) [34]. Participants will be instructed to train 5 days per week with a target of 24 levels per session (approximately 60 minutes). Cognitive training may be completed on a desktop computer, laptop, or tablet. A tablet device will be provided to any participants without access to a personal device for training. A customized training focus has been designed for this study based on areas of cognition known to be affected by COPD: attention, memory, speed of processing, and executive function [6-8]. These cognitive domains will be targeted using the following BrainHQ exercises: Double Decision, Hawk Eye, Visual Sweeps, Eye for Detail, Mind Bender, and Target Tracker [35,36]. The BrainHQ platform is designed to have training difficulty continuously adjusted to be performed “at threshold” of 70% to 80% accuracy, meaning that exercises become easier or more difficult based on the individual’s performance. All participants in the cognitive-physical training group will have a similar training session initially, but training parameters will become more individualized as users progress and the algorithm considers their individual performance history. Previous research indicates that the proposed training schedule (8 weeks, 40 hours total) is sufficient to elicit cognitive improvements [35,37,38].

### Data Collection

#### Primary Outcomes

##### Overview

The primary outcomes for this study include the following indicators of intervention feasibility: recruitment rate, retention, adherence, safety, and satisfaction. See Table 3 for an overview of the feasibility outcomes.

**Table 3:** Feasibility outcome measures.

Feasibility outcome	Assessment measure	Success criteria	Time point
Recruitment	Number of consented participants relative to the total number of eligible patients approached for consent	Recruitment rate of ≥30%	During study period
Retention	Number of participants who complete the final study assessment relative to the total number of participants enrolled and randomized	Retention rate of ≥80%	During study period
Adherence	Percentage of completion of physical and cognitive training intervention components according to training logs and BrainHQ records	Intervention adherence of ≥75%	During study period
Safety	Adverse events reported via exercise training logs and weekly check-in sessions	No study-related adverse events	During study period
Satisfaction	Participant satisfaction survey	≥80% reporting above-average or excellent satisfaction on a Likert scale	Weeks 1, 4, and 8

##### Recruitment

The recruitment rate will be defined as the percentage of consented patients relative to the total number of eligible patients who agreed to be approached by the study team for participation. Reasons for study participation and nonparticipation will also be collected. A recruitment rate of ≥30% has been established as our criterion to inform feasibility for a future study and help with modifications to recruitment for future studies.

##### Retention

Retention for the whole study and per group will be defined by the number of participants who complete the final study assessment compared with the number of participants enrolled and randomized. A retention rate of ≥80% (in each group) has been established as our criterion to determine feasibility for a future study.

##### Adherence

Adherence to the prescribed physical training and cognitive-physical training programs will be assessed via daily exercise training logs, the number of training levels completed in BrainHQ (displayed on a secure cloud-based group portal accessible by the study team), and weekly communications with participants. Adherence to the physical exercise training intervention will be expressed as the percentage of physical training days completed out of the total number of training days prescribed. Adherence to the cognitive training intervention will be expressed as (1) the number of cognitive training days completed out of the number of cognitive training days prescribed and (2) the number of cognitive training levels completed out of the total number of levels prescribed using the BrainHQ platform. Adherence to the interventions of ≥75% (ie, combination of physical training logs and cognitive training platform) is our criterion to determine feasibility for a future study.

##### Safety

Intervention safety will be monitored based on the occurrence and severity of adverse events. Participants will be instructed to log and report any side effects or adverse events during the intervention (ie, falls, injuries, and respiratory symptom worsening). Adverse events will also be reviewed by the exercise professional during weekly sessions.

##### Satisfaction

Participant satisfaction with the cognitive-physical training and physical training programs will be assessed at weeks 1, 4, and 8 using a Participant Evaluation Questionnaire developed for this study involving multiple-choice and open-ended questions. The results will be reviewed and analyzed by the study team at the end of the trial to determine overall satisfaction and any modifications required for future trials.

#### Secondary Outcomes

##### Overview

Effect size estimates will be evaluated using secondary outcome measures of physical and cognitive function, dual task performance, ADLs, and HRQL, as described in further detail in the following sections. These measures will be assessed at baseline and at 4 and 8 weeks after starting the intervention. See Table 4 for a summary of secondary outcome measures.

**Table 4:** Secondary outcome measures by time point.

Outcome		Assessment measure	Time points
**Characterization measures**			
	Sociodemographic information	Sociodemographic questionnaire	Baseline
	Cognitive screening	Montreal Cognitive Assessment Test	Baseline
	Disease impact	COPD^a^ Assessment Test	Baseline, week 4, and week 8
	Clinical characteristics	Medical chart review	During study period
**Physical function**			
	Lower-body strength	1-Minute Sit-to-Stand Test	Baseline, week 4, and week 8
	Lower-body function	Short Physical Performance Battery	Baseline, week 4, and week 8
	Aerobic endurance	Foot peddler endurance test	Baseline, week 4, and week 8
	Cognitive performance	BrainHQ assessment	Baseline, week 4, and week 8
	Dual-tasking performance	Foot peddler dual task test	Baseline, week 4, and week 8
**Self-reported outcomes**			
	Dyspnea	MRC^b^ Dyspnea Scale 18-item qualitative dyspnea scale Borg Dyspnea and RPE^c^ Scales	Baseline, week 4, and week 8
	Activities of daily living	The London Chest Activity of Daily Living scale	Baseline, week 4, and week 8
	Health-related quality of life	St. George’s Respiratory Questionnaire	Baseline, week 4, and week 8
	Mood	Depression, Anxiety, and Stress Scale (21 items)	Baseline, week 4, and week 8
	Exercise self-efficacy	Exercise Self-Regulatory Efficacy Scale	Baseline, week 4, and week 8
**Physical activity**			
	Physical activity habits	International Physical Activity Questionnaire–Short Form	Baseline
	Physical activity behaviors	Fitbit activity tracker	During study period
	Health care use	Medical chart review Self-reported	During study period and 1 year before study enrollment

^a^COPD: chronic obstructive pulmonary disease.

^b^MRC: Medical Research Council.

^c^RPE: Rating of Perceived Exertion.

##### Characterization Measures

###### Sociodemographic Information

Age, sex, ethnicity, employment status, educational level, and smoking history will be collected via chart review and self-report to characterize the sample and interpret findings from the cognitive assessments.

###### Montreal Cognitive Assessment

The Montreal Cognitive Assessment (MoCA) [39] is a 30-point screening test for mild cognitive impairment that has been previously applied in patients with COPD [12,40]. The MoCA specifically assesses short-term memory, visuospatial abilities, executive function, attention, concentration, working memory, language, and orientation to time and place. The MoCA will be administered only at the baseline assessment to help characterize the study population.

###### COPD Assessment Test

The COPD Assessment Test [41] is a short questionnaire for assessing and monitoring the impact of COPD. The COPD Assessment Test consists of 8 items that assess cough, the production of phlegm, chest tightness, breathlessness, activity limitation, confidence, sleep, and energy. Each item is rated on a 6-point scale, and the overall score ranges from 0 to 40, with a higher score indicating greater disease impact.

###### Clinical Characteristics

Clinical characteristics, including FEV_1_; medications (eg, inhaled medications, opioids, and prednisone); baseline 6-minute walk distance; Body Mass, Obstruction, Dyspnea, and Exercise index; and exacerbation history, will be ascertained from chart review up to 1 year before study enrollment. We will also capture anthropometric measures and BMI from pulmonary function tests if available from routine clinical evaluations. Comorbidities in our study participants will be assessed using the Charlson Comorbidity Index and through chart review using a comprehensive comorbidity questionnaire previously applied by our group [42].

##### Physical Function

###### Lower-Body Strength

The 1-Minute Sit-to-Stand Test [43] will be used to evaluate lower-body strength. This test involves participants completing as many sit-to-stand rises from a chair as possible in 1 minute. Participants will be instructed to stand fully upright and then sit down again immediately without delay, with arms crossed at the chest to avoid the use of the arms to rise from the chair. The research team will guide participants in selecting an appropriate chair (approximate height of 46 cm) with a flat, noncushioned bottom and no armrests. Participants will be prompted to report their Borg Dyspnea and Borg Leg RPE scores before and after the test.

###### Lower-Body Function

The Short Physical Performance Battery [44] will be used to assess lower-extremity performance, including balance (ability to stand with feet side by side, semitandem, and tandem), gait (usual-paced walking speed over 4 meters), and chair stand (time to rise from a chair 5 times). The Short Physical Performance Battery score is a summation of the scores on the 3 tests, each scored from 0 to 4, with a maximal cumulative score of 12.

###### Aerobic Endurance

Aerobic endurance will be measured using a foot peddler test developed by our research group for this study. Participants will pedal for as long as they can up to 15 minutes at a set speed of 50 revolutions per minute and a resistance setting that allows them to carry on a conversation (approximately 3-4 on the Borg RPE Scale). This intensity level is intended to be challenging but not exhausting. The primary endurance measure will be the number of minutes of testing completed. The reason for test termination will also be recorded. Oxygen saturation, qualitative dyspnea descriptors, Borg Dyspnea score, and Borg Leg RPE score will be measured before and after endurance testing. The complete aerobic endurance testing protocol is described in Textbox 1.

**Aerobic endurance foot peddler testing protocol.**

**Baseline measurements**

- Oxygen saturation with pulse oximetry, qualitative dyspnea descriptors, Borg Leg Rating of Perceived Exertion score, and Borg Dyspnea score will be measured before testing.

**Warm-up**

- Participants will pedal for 1 minute at a self-selected speed as a warm-up in order to become familiar with the foot peddler.

**Test period**

- Participants will continue pedaling at a set speed of 50 revolutions/minute and a resistance setting that allows them to carry on a conversation (approximately 3-4 on the Borg Rating of Perceived Exertion Scale) for as long as they can up to a maximum of 15 minutes. Participants will be prompted every few minutes to monitor their heart rate and oxygen saturation using an oximeter and their Borg Dyspnea score. The test will be stopped based on any of the following stopping criteria:
  15 minutes have been completed.
  Oxygen saturation drops to <85%.
  Self-reported Borg Dyspnea score is >7.
  The activity becomes unbearable (eg, severe leg pain or the participant is too tired to continue).
  Heart rate reaches 85% of age-predicted maximum (calculated as 220 beats/minute–age).
  Participant is unable to maintain a speed of 50 revolutions/minute.
  The research team member decides to stop the test because of apparent breathlessness or discomfort of the participant.

**Posttest measurements**

- The reason for test termination will be recorded, and repeat measurements will be taken for oxygen saturation, qualitative dyspnea descriptors, Borg Leg Rating of Perceived Exertion score, and Borg Dyspnea score. We will also ascertain if participants have any additional comments regarding their test performance.

**Cooldown**

- Participants will be instructed to continue pedaling at a self-selected speed and resistance to cool down until heart rate drops to <110 beats/minute.

##### BrainHQ Cognitive Assessment

Cognitive performance will be evaluated via the BrainHQ assessment platform. A custom assessment battery modeled on the training exercises will be used, with a focus on evaluating changes in performance from training as a measure of target engagement [45]. These assessments were chosen in consultation with the scientific team at BrainHQ and coinvestigators with cognitive expertise (DG, RG, and JLC).

##### Dual Task Performance

Dual task performance will be evaluated through a dual task test on the foot peddler, as previously undertaken in other populations [46,47]. Participants will complete a motor, cognitive, and combined dual task (all of 1-minute duration) in random order: (1) the *cognitive task* involves spelling 5-letter words backward from a list of 100 words provided verbally (homonyms excluded), (2) the *motor task* involves pedaling on the foot peddler at a set speed of 50 revolutions per minute and a self-selected resistance corresponding to a moderate-intensity level allowing participants to hold a conversation (approximately level 4 on the Borg RPE Scale), and (3) the *dual task* involves spelling and pedaling at the same time. Participants will be instructed to prioritize both tasks equally. Spelling tasks (single and dual tasks) will be audio recorded using a Dictaphone to allow for precise scoring. The number of words correctly spelled backward and the distance covered while pedaling will be compared between the single and dual task conditions to calculate a dual task cost score for both the cognitive and physical performance tasks.

##### Patient-Reported Outcomes

###### Medical Research Council Dyspnea Scale

The Medical Research Council Dyspnea Scale [48] will be used to assess the degree of functional disability because of breathlessness. Participants will indicate the extent to which breathlessness limits their daily physical activities on a scale from 1 (only breathless with strenuous activity) to 5 (too breathless to leave the house or breathless when dressing or undressing).

###### ADL Outcomes

The London Chest Activity of Daily Living scale [49] will be used to assess limitations in performing ADLs because of dyspnea. It comprises 15 questions across 4 domains: self-care, domestic activities, physical activities, and leisure. Each question is rated on a scale from 0 to 5, with 5 representing the greatest dyspnea-related impairment. Total scores range from 0 to 75 points, with higher values indicating greater ADL limitations [50]. The London Chest Activity of Daily Living scale has been applied in patients with COPD with a minimally important difference of 4 points [49].

###### HRQL Outcomes

The St. George’s Respiratory Questionnaire [51] will be administered to measure the impact of COPD on overall health, daily life, and perceived well-being. The questionnaire comprises 50 items. Scores range from 0 to 100, with higher scores indicating worse HRQL. A minimally clinically important difference of 4 points has been identified in patients with COPD [52].

###### Mood

The Depression, Anxiety, and Stress Scale–21 [53] is a 21-item questionnaire that measures the emotional states of depression, anxiety, and stress. It has acceptable to excellent internal consistency and normative data for the COPD population [54]. Scores are categorized as normal, mild, moderate, severe, and extremely severe for each of the 3 domains.

###### Self-Efficacy to Exercise

The Exercise Self-Regulatory Efficacy Scale (Ex-SRES) [55] will be administered to measure exercise self-regulatory efficacy among participants. The Ex-SRES consists of 16 items asking participants to indicate the degree to which they are confident that they could continue to exercise regularly (3 times/week for 20 minutes) when faced with various barriers. Ratings are provided from 0% (not at all confident) to 100% (highly confident). The validity and reliability of the Ex-SRES have been demonstrated in individuals with COPD [55].

##### Physical Activity Levels

The *International Physical Activity Questionnaire–Short Form* [56] will be used to characterize baseline physical activity levels among participants. The International Physical Activity Questionnaire–Short Form consists of 7 questions that capture average time spent sitting, walking, and engaging in moderate and vigorous physical activity over the previous 7 days to provide an estimate of overall activity levels based on metabolic equivalent of task minutes per week.

During the 8-week study period, physical activity levels will be measured via a wrist-worn activity tracker (Fitbit Charge), which will capture daily step count, sedentary time, and number of active minutes (minutes of activity classified as “moderate” or “vigorous” intensity based on heart rate) [57].

##### Health Care Use

Health care use over the course of the 8-week intervention and 1 year before study enrollment will be assessed through chart review. The following information will be collected: COPD exacerbation history, history of falls, emergency department visits, admissions to hospital (reasons and length of hospital stay), and mortality during the study period.

### Statistical Procedures

#### Sample Size

A sample size of 12 participants per group is considered appropriate for a pilot study to assess feasibility [58], accounting for an estimated attrition rate of approximately 20% in each group. Power calculations were not performed for the secondary outcomes as the primary study purpose is to assess effect estimates for further program development and evaluation in a future RCT.

#### Data Analysis

Descriptive statistics will be used to summarize recruitment and retention rates, program adherence, participant satisfaction, and safety in both groups. Means and 95% CIs will be reported for the secondary outcomes at baseline, 4 weeks, and 8 weeks within and between groups to derive effect estimates for analysis and sample size calculations for a subsequent larger-scale RCT. Paired 2-tailed *t* tests and 2-tailed *t* tests will be used to compare the changes in the secondary outcome measures over the 8-week study period within and between the 2 randomized groups, respectively. All participants will be included in the analyses based on the intention-to-treat principle according to their group allocation from randomization.

We will explore the contribution of age and sex to feasibility end points and secondary outcomes. Given the modest sample size and importance of age for both cognitive and physical function, we limited our inclusion criteria to the age of ≥50 years to ensure a more homogenous study population. Other important clinical factors such as BMI and medications will be included for descriptive purposes.

### Data Quality and Management

Our research team is trained in the study processes, measurement protocols, and institutional requirements. We have received peer review comments from the Canadian Lung Association (Multimedia Appendix 1) and incorporated this feedback into the study protocol. Standard operating procedures are in place for all study procedures. The research team will make every effort to have participants complete all study elements, and incomplete or missing data will not be imputed. All participants will be closely supervised by the exercise professional, who will support adherence and accurate data collection.

As per national and institutional research guidelines, all participants will be given a unique study ID to be used in place of their name for all study data. Physical data collection forms and data collected from questionnaires will be kept in a cabinet in the principal investigator’s office under lock and key. Electronic data will be stored on a password-protected research server. Once data collection is complete, deidentified data will be exported to statistical software for analysis.

## Results

Recruitment began in January 2022. It is estimated that the study period will be 30 months long, with data collection to be completed by December 2023. Study results will be submitted for publication in the summer of 2024.

## Discussion

### Study Summary

COPD is associated with several physical and cognitive impairments contributing to limitations in daily activities that often involve the simultaneous performance of a physical (eg, walking) and cognitive (eg, talking) task, known as dual tasking. The benefits of pulmonary rehabilitation in reducing COPD symptoms and improving daily function are well established [21], and it is known to be feasible and effective when delivered remotely in the home environment via telehealth [26,27,31]. However, traditional pulmonary rehabilitation programs typically focus mainly on physical exercise training without addressing cognitive function despite evidence that many patients with COPD have some cognitive limitations, which can contribute to greater functional limitation, increased health care use, and decreased HRQL [6,23]. Patients with COPD with cognitive limitations are also at increased risk of noncompletion of pulmonary rehabilitation [59], suggesting that a better understanding of cognitive limitations and approaches to improve cognition may lead to better adherence and outcomes. However, to date, no studies have examined a pulmonary rehabilitation approach that specifically combines both physical and cognitive training. Therefore, this protocol describes a pilot RCT that is entirely internet based and home based and combines cognitive and physical training in patients with COPD with the aim of further improving daily function and HRQL beyond traditional pulmonary rehabilitation approaches.

### Cognitive Training Mechanisms

Cortical activity is essential for all motor performance, ranging from complex activities to basic ADLs; thus, the benefits derived from cognitive training can be obtained even in those with normal cognition [60]. Cognitive training has been shown to be more effective in those completing a physical exercise intervention as the neural network is primed for neuroplasticity. Thus, cognitive training may be a complementary strategy in conjunction with physical exercise to improve dual tasking in patients with COPD, which is often required during routine daily activities (eg, talking or avoiding obstacles while walking). COPD is associated with impaired dual-tasking performance, manifested as changes in dual task gait properties, including reduced gait speed [12] and increased gait variability [15]. There is also evidence from our group that loss of neural automaticity may be an underlying mechanism contributing to slower gait speed among individuals with COPD [11]. Collectively, these changes suggest that cognitive limitations may arise from increased attentional demands and neural inefficiency, which may contribute to decreased cognitive and physical performance in COPD [17]. Although traditional pulmonary rehabilitation does not appear to improve dual task gait in patients with COPD [15], cognitive training is known to produce distinct benefits on dual task reaction time [61] and has been shown to independently improve measures of functional mobility among healthy older adults [62]. Therefore, the addition of cognitive training may help improve neural efficiency and attentional capacity in patients with COPD, leading to improvements in dual-tasking ability that may translate to better performance in daily activities and HRQL and reduction in the risk of falls.

Clinical practice guidelines established by several leading organizations recommend cognitive training as a strategy to decrease the risk of cognitive decline (World Health Organization; American Academy of Neurology; National Academies of Sciences, Engineering, and Medicine; and Agency for Healthcare Research and Quality) [63-66]. These recommendations were largely established based on strong scientific evidence in the form of meta-analyses showing both near and far transfer effects from cognitive training in older community-dwelling adults, those with mild cognitive impairment, and those with dementia [67-71]. BrainHQ by Posit Science is a computerized cognitive training platform offering game-based exercises that target the speed and accuracy of information processing across the domains of attention, memory, spatial navigation, executive function, and emotional processing. In the largest RCT on cognitive training to date involving 2832 healthy older adults, speed-of-processing training on BrainHQ was found to improve cognitive and functional performance to a greater extent than other forms of memory or reasoning training, which translated to a reduction in risk of impairments in daily activity performance and an increase in reported quality of life that remained at follow-up of 5 to 10 years [72]. BrainHQ training resulted in improved visual processing speed and accuracy, and these improvements were associated with improved balance and smaller decrements in dual task gait speed among older adults [73,74], which has important implications for patients with COPD who are at risk of falls [42]. To our knowledge, this study will be the first to explore BrainHQ cognitive training in a population of individuals with chronic lung disease.

Given the breadth of impairments associated with COPD and the complexity of routine daily activities, the rehabilitation approach described in this paper combining physical exercise with cognitive training targeting attention, memory, executive functioning, and speed of processing may offer the flexibility to further improve daily function and reduce the risk of falls in patients with COPD beyond physical training alone. Notably, the cognitive training modality to be implemented in this study is distinct from the proposed dual task outcome measure, supporting the potential transfer of gains to a variety of daily activities.

### Strengths

There are several strengths to this study. The physical training program and assessments were specifically designed to support feasibility and safety in the home environment. All participants will be provided with exercise training equipment (foot peddler, resistance bands, Fitbit, and pulse oximeter), which will improve safety and accessibility and standardize physical training and assessments. This study is novel as it provides a new approach to remote assessments of aerobic endurance (ie, foot peddler), functional outcomes, and cognitive assessment and training as part of a home-based pulmonary rehabilitation program for patients with COPD [27]. Our protocol may optimize not only accessibility but also outcomes in home-based pulmonary rehabilitation programs. The use of commercially available software programs (Fitbit, Physiotec, and BrainHQ) supports the future clinical translation of this work as these and other similar programs may be used by clinicians and patients looking to integrate cognitive-physical training into pulmonary rehabilitation programs. Furthermore, the chosen cognitive training platform (BrainHQ) has the largest evidence base and, to our knowledge, is the only brain training program that meets all the National Academy of Medicine criteria for cognitive training [29,30]. Our specific cognitive training protocol was designed in consultation with neuropsychology and cognitive science experts to specifically target common areas of deficit among individuals with COPD.

### Limitations

We also acknowledge that there are limitations to the design of this study. Given the small sample size, we will be unable to adjust the analyses for potential confounders such as medications (opiates, sedatives, or corticosteroids) or COPD exacerbations; however, these factors will be tracked throughout the study period to explore their influence on selected outcomes. This study will be conducted entirely remotely, and therefore, we are unable to prospectively measure specific tests of outcomes such as pulmonary function (ie, spirometry) or neural activity (ie, functional near-infrared spectroscopy). However, some of this information will be derived from chart review or will require future study. Furthermore, cognitive assessments will be completed independently at home, and differences in device or screen size (ie, computer vs tablet) may influence the results. Participants who do not have access to a suitable device will be provided with a tablet for the duration of the study. Owing to the nature of the training intervention and internet-based environment, blinding of participants and the exercise professional is not possible, thus creating a risk of bias. Furthermore, most of the physical training measures and intensity will be ascertained through self-report, which may not be as accurate as if they were captured electronically from the training devices.

### Conclusions

A partly supervised, home-based, telehealth cognitive-physical training program may be an accessible intervention to improve cognitive function and further address dual-tasking abilities in people living with COPD. Evaluating the feasibility and effect estimates is a critical first step to inform future clinical trials evaluating this approach and its effects on physical and cognitive function, ADL performance, and HRQL.

## Appendix

Multimedia Appendix 1 Peer review report from the Chronic Obstructive Pulmonary Disease Catalyst Grant Award.

## Abbreviations

ADL: activity of daily living
COPD: chronic obstructive pulmonary disease
Ex-SRES: Exercise Self-Regulatory Efficacy Scale
FEV1: forced expiratory volume in the first second
HRQL: health-related quality of life
MoCA: Montreal Cognitive Assessment
RCT: randomized controlled trial
RPE: Rating of Perceived Exertion
UHN: University Health Network
