# Supplementary material for: Feasibility of a Home-Based Cognitive-Physical Exercise Program in Patients With Chronic Obstructive Pulmonary Disease: Protocol for a Feasibility and Pilot Randomized Controlled Trial
Source: JMIR Res Protoc. 2023 Jul 12;12:e48666. doi: 10.2196/48666 (PMC10372770; doi:10.2196/48666)
Supplement: Multimedia Appendix 1 [file resprot_v12i1e48666_app1.pdf]

The Lung Association – National Office
L'Association pulmonaire – Bureau National
502-885 Meadowlands Drive, Ottawa, Ontario K2C 3N2
T 613-569-6411 | F 613-569-8860 | info@lung.ca | **lung.ca**
Lung Health Information Line 1-866-717-2673

Breathing as One - Boehringer Ingelheim Canada/CIHR-ICRH COPD Catalyst Grant Award

Principal Investigator	Dmitry Rozenberg
Project Title	Feasibility of a Home-Based Cognitive-Physical Exercise Program in Chronic Obstructive Pulmonary Disease Patients: A Pilot Randomized Control Trial

Summary Notes

Strong team with different skill sets. Novel and interesting intervention that is doable in this time frame. Some concerns about possible attrition and not getting the signal needed. Would like to see a discussion of confounders. Pre- to post-intervention will be hard. Consider obtaining more qualitative data from users for balance. Could lead to an interesting study.

Primary Reviewer's Comments

Applicant(s)

This is as very strong team including experts in rehabilitation sciences, pulmonary rehabilitation, respirology, psychology, and physiotherapy. The nominated PI, Dr. Rozenberg, is a PhD/MD and is a respirologist and clinical scientist and pulmonary rehab director of lung transplant in the University Health Network at University of Toronto since 2018. He has a strong track record of funding and holds a Professorship in Rehabilitation for 500K and notably a $30K Canadian Donation and Transplantation research program grant for feasibility of home-based program in liver and lung transplant recipients. He demonstrates a lot of involvement in knowledge translation activities and community and volunteer engagement. Dr. Rozenberg has 29 publications and one book chapter in the last 5 years, most in moderate impact medical and thoracic journals, of which he is first or senior author on 10. He has 19 papers in 2020, which shows an impressive trajectory of productivity.

The rest of the team includes Drs. Reid (Co-PI), Santa Mina, Campos (CRC chair in Multisensory Integration and Aging), and Stanbrook, who have considerable track records of funding, publication, supervision, and mentorship, as well as expertise in elements of training and function testing, measurement of cognitive function, home based exercise training, and COPD management.

Proposed Research Project

The Lung Association – National Office
L'Association pulmonaire – Bureau National
502-885 Meadowlands Drive, Ottawa, Ontario K2C 3N2
T 613-569-6411 | F 613-569-8860 | info@lung.ca | lung.ca
Lung Health Information Line 1-866-717-2673

A strong rationale is presented for the study where evidence is provided for both cognitive and physical impairments in COPD and how traditional COPD management strategies have primarily focused on treating the physical limitations. They also present a compelling justification for how cognitive limitations can result in decreased physical performance when they are performed simultaneously (i.e. dual-task interference). The applicants hypothesize that a training modality targeting several cognitive domains accompanied by physical training may offer the desired flexibility and testing effects on daily task performance in COPD patients. For this pilot RCT study they will address two specific aims:

1) To evaluate the feasibility (recruitment rate, program adherence, attrition, safety, and participant satisfaction) of an 8-week home-based C-PT program in COPD patients.

2) To derive preliminary estimates on intervention efficacy with C-PT on dual-task interference, cycling performance, balance, ADL, and HRQL.

Strengths of the Proposed Study
- The study is novel and the combination of cognitive and physical training is interesting and potentially clinically important if it can be effectively administered.

- Team is very strong and appears to have the access, experience, and training necessary to recruit patients, perform the primary and secondary outcomes, and deliver the home-based exercise and cognitive therapy.

- The intervention is interesting and novel (albeit quite a commitment if they are in the combined group), the program is being delivered by exercise professionals but patients having access to exercise videos and COPD-specific manuals is well thought out and could help patients considerably.

- As addressed by the applicants, the study can be performed during the COVID pandemic and could be even more important considering some patients may have been more inactive than normal over the last year.

Weaknesses and Limitations of the Proposed Study
- This is fundamentally a phase III RCT design, comparing usual care (home-based exercise rehabilitation) with usual care + cognitive therapy being proposed as a feasibility and data-generating study. I understand why the applicants have taken this approach (they want to know the effect of cognitive therapy in addition to the physical training compared to physical training alone) but whether they will get much of a signal with potentially only 9 finishing (12 per arm with the potential for 20% attrition) is a concern. My issues in this regard are 1) the heterogeneity of the patient sample in each group, 2) there will be responders and non-responders in both arms, and 3) the effects of cognitive therapy on top of a physical training

The Lung Association – National Office
L'Association pulmonaire – Bureau National
502-885 Meadowlands Drive, Ottawa, Ontario K2C 3N2
T 613-569-6411 | F 613-569-8860 | info@lung.ca | lung.ca
Lung Health Information Line 1-866-717-2673

intervention on backward spelling (when the participants are not directly practicing this skill) might be quite small (although I admit there is no way of knowing this). As such, they may not get enough of an effect to accurately power a future study even though the study will be deemed feasible.

- The sample will be stratified for sex but with such a small sample size, large differences in groups for baseline exercise capacity and cognitive function could greatly influence the study findings. How will these important cofounders be taken into consideration?

- One of the secondary measures mentioned is a cycling test while reading backwards. However, no details of this test are presented. On a foot peddler with the patient at home potentially on their own, how will this test be performed, how will intensity be set, and how will the measurements be standardized (across the three time points) and monitored?

- It is not mentioned whether the person performing the cognitive and exercise assessments at each time point will be blind to the study allocation. In the budget, funding is requested for the RA to train and virtually assess the participants. If the same person does both, this will greatly bias the data.

- Recruiting, training, and assessing 24 individuals with COPD through an 8-week intervention in 9 months is highly challenging and the timeline is ambitious. Confidence in the feasibility of the study could have been strengthened by providing evidence to support previous studies of this nature that have been completed in a similar timeline. Additionally, accrual rates specific to this group's research for previous interventional studies in COPD would have strengthened this aspect of the proposal.

Impact on COPD Research

It is hoped the study will provide preliminary data of the feasibility and efficacy of combined physical and cognitive therapy to power a future RCT. If that future study is successfully funded and performed, positive findings could change how we manage patients with cognitive and physical impairments associated with COPD. I have no additional issues with this study trying to generate preliminary data for a future RCT, which will be important. However, it is unlikely to lead to a meaningful publication on the efficacy aspect of the project due to the comments made regarding the very small sample size for a RCT assessing an exercise intervention + cognitive therapy versus an exercise intervention alone.

Knowledge Translation Plan

A relatively strong knowledge translation plan is presented. The applicants will use national and international platforms (i.e. ATS, CTS) to present the findings of the study, they will also

The Lung Association – National Office
L'Association pulmonaire – Bureau National
502-885 Meadowlands Drive, Ottawa, Ontario K2C 3N2
T 613-569-6411 | F 613-569-8860 | info@lung.ca | lung.ca
Lung Health Information Line 1-866-717-2673

disseminate findings through the diverse professional networks of the study team members. The ATS web committee platform, social media, conference presentations, and traditional journal publications will also be utilized. It is mentioned that they will have two patients involved to help implement the research and facilitate transition across COPD working groups, but it is not really clear how this will be specifically done.

Budget Considerations

Generally, the budget items requested seem appropriate. The applicant is asking for money toward a Kinesiology research assistant, exercise equipment, fitbits, tablets, etc. While the RA is justified and it is fairly obvious how the equipment will be used, it is not justified within the context of the study. Additionally, 20 hours of statistician support ($2000) and 24 x $25 for ECGs from lifelabs are requested but not justified or mentioned in the proposal.

A grant for $150K is submitted at time of application to the University Health Network / Mount Sinai for what appears to be the same project in COPD and ILD is mentioned and addressed. However, in the PI's CV it is mentioned that they have funding for home-based research in Lung Tx. Overlap between this study and other funds held are not justified.

Ethics Considerations

I have no ethical concerns with the study. The ethics certificate from the institutional REB still needs to be sent in at the time of application.

Summary Comments

This is a strong team proposing an interesting and novel study. The proposed intervention could be highly beneficial for individuals with COPD and impaired cognitive function. If a large effect is seen from the addition of cognitive therapy I believe the idea has the potential to lead to a successful CIHR application. However, the proposed project is ambitious within a one-year timeline and there is the potential that a larger initial study is required to obtain the preliminary data needed.

Secondary Reviewer's Comments

Applicant(s)

This team of five had a good mix of early, mid-career, and senior researchers. The team is also interprofessional, which will strengthen the research process. The PI is an MD PhD, early career researcher. As the Director of Lung Transplantation, it seems there would be a solid network for KT and dissemination as well as other collaboration if/as needed. The co-PI is a

The Lung Association – National Office
L'Association pulmonaire – Bureau National
502-885 Meadowlands Drive, Ottawa, Ontario K2C 3N2
T 613-569-6411 | **F** 613-569-8860 | info@lung.ca | **lung.ca**
Lung Health Information Line 1-866-717-2673

physical therapist with an expansive knowledge. These two people will contribute 10% to the project. There is not a lot of detail on interaction of team (meetings? communication?), and it is difficult to assess how the team will work together.

The PI has an excellent track record in research and publications.

Dr. Campos, as CRC chair, is a strong addition to the team.

Proposed Research Project

The proposed project will compare cognitive + physical training to physical training alone in an 8-week home-based program for people with COPD. They will build off their own previous research and have also submitted a similar proposal for funds.

The proposal notes: "Dual-task cognitive and physical training has not been performed in people living with COPD despite the fact that cognitive impairment has been well described for several decades." This is a novel addition to the literature. This study will focus on 2 aims: 1) to evaluate feasibility of the program, and 2) to derive preliminary estimates on the efficacy of the intervention. These objectives seem fair and feasible within the proposed timeline.

This pilot RCT will randomize 24 patients to receive either PR+ cognitive training or just PR. The plan for both seems to be sound and based on solid evidence.

Concerns about recruitment and COVID were discussed. Concerns about comparisons among the two groups were also discussed, but not convincingly. Given the possible confounding factors (from current Rx to current exercise knowledge), it is not clear that 24 participants would be sufficient. That said, the team aptly describes this as a feasibility pilot only and are aware of these limitations.

STRENGTHS
- Novel
- Strong team (good access to conduct research, strong knowledge base and interprofessional team)
- Doable within the timeframe

WEAKNESSES
- Design may not get the necessary results; stratified by sex, usual care of PT vs. PT + cognitive therapy.
- Will 24 be sufficient with 20% attrition? They may not get data needed for future RCT.
- The effects of CT might be quite small because of confounding variables.

The Lung Association – National Office
L'Association pulmonaire – Bureau National
502-885 Meadowlands Drive, Ottawa, Ontario K2C 3N2
T 613-569-6411 | F 613-569-8860 | info@lung.ca | **lung.ca**
Lung Health Information Line 1-866-717-2673

Impact on COPD Research

Seems very exciting and easy to add something like this one. I do wonder about the feasibility in terms of cost – is there a cost-benefit to providing extra exercises? Could this be accomplished in a different way? Who would fund this added service in the future?

Knowledge Translation Plan

Plan follows traditional academic KT including journals (x2) and conference presentation. Also noted the network of the research team as a means to distribute findings. They will also make use of social media through established sites like ATS and ETS.

Budget Considerations

Seems appropriate, though there is a significant amount for technology/equipment that was not justified/explained.

RA is doing significant amount of work – not sure if enough hours were provided/allotted.

NOTE: Other funding being applied for indicated a 50% overlap.

Ethics Considerations

Involves human subjects. Proposal indicates ethics form needed to be sent.

Other Considerations

The proposal mentions patients in the KT plan ("We will also aim to have 2 COPD patient partners to help with research implementation and facilitate translation across COPD working groups"), but there are no further details nor a line item in the budget for patients.

Summary Comments

Overall well written and clear. Involvement of patients seems only cursory. Would be excellent to understand patient perspective after the intervention (i.e. adding qualitative methods of some sort, though I appreciate this may be out of scope and expertise).
